# Lack of Social Support Raises Stress Vulnerability in Rats with a History of Ancestral Stress

**DOI:** 10.1038/s41598-017-05440-8

**Published:** 2017-07-13

**Authors:** Jamshid Faraji, Nabiollah Soltanpour, Hamid Lotfi, Reza Moeeini, Ali-Reza Moharreri, Shabnam Roudaki, S. Abedin Hosseini, David M. Olson, Ali-Akbar Abdollahi, Nasrin Soltanpour, Majid H. Mohajerani, Gerlinde A. S. Metz

**Affiliations:** 1 Golestan University of Medical Sciences, Faculty of Nursing & Midwifery, Gorgan, I. R. of Iran; 2 University of Lethbridge, Canadian Centre for Behavioural Neuroscience, Lethbridge, Canada; 3 Babol University of Medical Sciences, Department of Anatomical Sciences, Babol, I. R. of Iran; 4 Islamic Azad University, Department of Psychology, Tonekabon Branch, Tonekabon, I. R. of Iran; 5 Avicenna Institute of Neuroscience, Department of Behavioural Studies, Yazd, I. R. of Iran; 6 Golestan University of Medical Sciences, Department of Anatomy, Gorgan, I. R. of Iran; 7 University of Alberta, Department of Obstetrics and Gynecology, Edmonton, Canada

## Abstract

Stress is a primary risk factor for psychiatric disorders. However, it is not fully understood why some stressed individuals are more vulnerable to psychiatric disorders than others. Here, we investigated whether multigenerational ancestral stress produces phenotypes that are sensitive to depression-like symptoms in rats. We also examined whether social isolation reveals potentially latent sensitivity to depression-like behaviours. F4 female rats born to a lineage of stressed mothers (F0-F3) received stress in adulthood while housed in pairs or alone. Social isolation during stress induced cognitive and psychomotor retardation only in rats exposed to ancestral stress. Social isolation also hampered the resilience of the hypothalamic-pituitary-adrenal axis to chronic stress and reduced hippocampal volume and brain-derived neurotrophic factor (BDNF) expression. Thus, synergy between social isolation and stress may unmask a latent history of ancestral stress, and raises vulnerability to mental health conditions. The findings support the notion that social support critically promotes stress coping and resilience.

## Introduction

Recent evidence has shown that the susceptibility to many complex diseases, such as psychiatric disorders, is causally linked to a history of transgenerational adverse experiences^1–4^. While ancestral stress may promote depression-like states in generations of offspring^5–7^, chronic stress during a lifetime in particular is a recognized risk factor for depression^8, 9^. Accordingly, experience of stress may be linked to the major hallmarks of affective disorders, such as low mood, anhedonia and loss of motivation^10^, reduced hippocampal volume^11–13^ and diminished neurogenesis in dentate gyrus^14^. In turn, stress resiliency, coping and superior therapeutic response in mental health disorders are associated with larger hippocampal volume^15, 16^.

The endocrine and emotional consequences associated with stress may also occur in the offspring^2, 17, 18^. In fact, stress can generate heritable epigenetic signatures linked to changes in affective behaviour that propagate to subsequent generations^19^. A recent study indicated that cumulative impact by multigenerational prenatal stress may generate new behavioural traits in the F4 generation^20^. Both prenatal and multigenerational stress re-program hypothalamic-pituitary-adrenal (HPA) axis function and may result in increased sensitivity to stress in later life^21, 22^. Thus, the response to stress in an individual with ancestral stress may be exacerbated^23^. Any latent susceptibility to psychological disturbances may therefore be exacerbated by stress in adulthood^24^. According to the double-hit hypothesis^25, 26^ one would expect that the cumulative impact of stress across generations and across a lifetime unmasks a latent vulnerability to affective disorder.

Here, we proposed that social isolation during stress in adulthood could enhance vulnerability to depression-like outcomes in rats with a history of multigenerational ancestral stress. In turn, we hypothesized that social support will promote stress resiliency in a lineage of vulnerable rats. Stress vulnerability was generated by multigenerational exposure to prenatal stress. The result of our experiment suggest that recurrent prenatal stress across generations causes latent (silent) stress vulnerability that becomes overt by interaction of two different stressors in later life, social isolation and restraint stress. Hence, postnatal adversities may synergistically interact to unfold their psychopathological impact on a vulnerable population.

## Materials and Methods

### Animals

One hundred three female Wistar rats (2–3 months of age; 230–300 g), bred and raised at the local vivarium, were used in this study. All animals were maintained on a 12-hour light/dark cycle (lights on at 7:30) with *ad libitum* access to food and water. All generations (F0-F3) excluding fourth-generation (F4) rats were paired with a male for one hour per day until mating occurred. Pregnancy of the rats was confirmed by weight gain 10–12 days later. Pregnant rats were then housed individually from gestational day 19 until delivery. Prior to the testing, F4 rats were handled for approximately 5 min daily for five consecutive days for habituation to the experimenters. Body weight in all animals was monitored throughout the experiment. All procedures in this study were carried out in accordance with the National Institute of Health Guide to the Care and Use of Laboratory Animals and were approved by the Avicenna Institute of Neuroscience Animal Care Committee (#900-55108).

### Experimental Design

**Figure 1:**
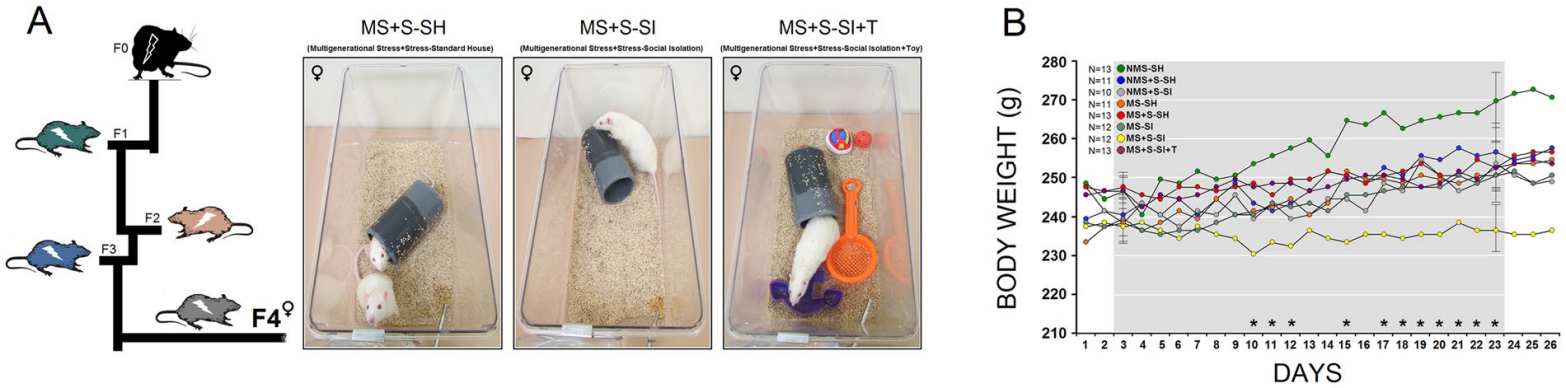
(**A**) *Experimental design to induce multigenerational and adulthood stress in F4 females*. Left: Animals of the parental generation (generation 0; F0) and their prenatally stressed pregnant daughters (filial generations 1–3; F1-F3) also received stress during late gestation. Right: the F4 generation that originated from a stressed maternal lineage received a stress challenge in adulthood while housed in standard paired housing conditions (MS + S-SH), socially secluded by singular housing (MS + S-SI), or received environmental enrichment with toys while being housed alone (MS + S-SI + T). (**B**) *Stress and social isolation limit weight gain in ancestrally stressed rats*. Grey-square zone indicates the 21-day stress period. Only MS + S-SI and MS + S-SI + T groups failed to gain weight during stress. A significant difference was observed between NMS-SH and MS + S-SI groups on the last day of stress exposure. Asterisks indicate significant difference between NMS + S-SI and MS+S-SI groups: *p ≤ 0.05. Error bars show ± SEM.

The multigenerational stress model involved exposing pregnant rat dams of the parental generation (F0), their pregnant daughters (F1), granddaughters (F2) and great-granddaughters (F3) to daily restraint stress during gestational days 11–18. The multigenerational stress lineage, therefore, was produced by stressing pregnant mothers in 4 consecutive generations (F0-F3) to produce the stressed F4 generation. Prior to testing, the F4 animals were randomly assigned to one of the following five experimental groups: (1) Multigenerational Stress-Standard Housing (MS-SH), *N* = 12: rats born to the multigenerational stress lineage were housed in pairs; (2) Multigenerational Stress + Stress-Standard Housing (MS + S-SH), *N* = 14: multigenerationally stressed rats received restraint stress in adulthood and were housed in pairs during the stress period. (3) Multigenerational Stress-Social Isolation (MS-SI), *N* = 12: multigenerationally stressed rats were housed alone at the time when other groups received stress in adulthood; (4) Multigenerational Stress + Stress-Social Isolation (MS + S-SI), *N* = 14: multigenerationally stressed rats received restraint stress in adulthood and were housed alone during stress period; (5) Multigenerational Stress + Stress-Social Isolation + Toys (MS + S-SI + T), *N* = 12: multigenerationally stressed rats received restraint stress in adulthood and were housed alone along with toys in their cages during the stress period in adulthood (Fig. 1A). Three additional generationally-matched groups served as controls: (1) Non-Multigenerational Stress-Standard Housing [F4; NMS-SH; *N* = 12] and (2) Non-Multigenerational Stress + Stress-Standard Housing [F4; NMS + S-SH; *N* = 14], and (3) Non-Multigenerational Stress + Stress-Social Isolation [F4; NMS + S-SI; *N* = 13].

### Gestational Stress

Timed-pregnant rats (F0-F3) were stressed daily from gestational days 11 to 18 by restraint. Restraint of the body for 60 min in a transparent Plexiglas cylinder (11 cm inner diameter; Scientific Aid Inc., Tabriz, Iran) was given daily in alternating intervals in the morning (between 9:00–11:00 hours) and afternoon (between 15:00–17:00 hours), as modified from earlier descriptions^27^.

### Adulthood Stress

Stress in adulthood was induced by restraint for 60 min for 21 consecutive days, as described above. For restraint stress, F4 female animals (on average 2.17 months old) in the stress groups (i.e. NMS + S-SH, on average 2.12 months old; NMS + S-SI, 2.27 months old; MS + S-SH, 2.18 months old; MS + S-SI, 2.05 months old; and MS + S-SI-T, 2.21 months old) were maintained in custom-made transparent Plexiglas tubes (9 cm inner diameter) of adjustable length. The tubes allowed the complete restraint of the animals while at the same time allowing them to breathe through perforated ends of the tube. The tubes maintained the animals in a standing position without compression of the body. All stress rats were stressed simultaneously in a quiet room with approximately 100 cm distance between restraint tubes. Control animals were transported to a room near the stress room but remained undisturbed in their home cages during the stress duration. All animals including controls received equal amounts of handling daily throughout the experiments.

### Blood Samples and Corticosterone Assays

All rats in this experiment underwent blood sampling before and during stress in adulthood. The procedure for blood sampling was the same as previously reported by Faraji and others^28^. Blood samples were taken at three time points: two days prior to restraint stress (pre-stress), first day of stress when restraint stress was terminated (post-stress1; acute time point), and one day after the stress period was completed (day 22; post-stress2; chronic time point). Rats were placed in a restraint tube and blood samples were obtained by tail notch with a scalpel blade. Pre-stress blood was collected from all rats within the first 1–2 min of being placed in the tube to ensure circulating corticosterone (CORT) levels did not have the chance to significantly increase in response to the brief stress of the procedure. Acute and chronic time blood samples were collected using the same procedure. The same sampling procedure was applied to stress rats on the two time points while they were still in the tubes. All blood samples (0.5–0.6 mL) placed in heparinized tubes were then transferred to centrifuge tubes and plasma was obtained by centrifugation at 8000 rpm for 8 min. The plasma samples were stored at −20 °C until analyzed for CORT concentration using commercial radioimmunoassay kits (Salimetrics, UK). All samples were collected in the morning hours between 9:00 and 11:00 hours. The intra- and inter-assay coefficient of variations (CV) in the present study were 9.1% and 11.8%, respectively. No behavioural testing was performed on blood sampling days.

### Behavioural Procedures: Morris Water Task (MWT)

The hidden platform version of the MWT (155 cm diameter) was used to assess spatial performance^29^. Animals (*N* = 6–9) were tested by a one-day testing protocol (12 trials per animal) 2 days after stress in the morning (between 8:00 and 11:30 hours). Animals were taught to escape from the water (21 ± 1 °C) by climbing onto the hidden platform (12 cm diameter). Each trial began with the rat being placed in the pool at one of the four cardinal compass positions around the perimeter of the pool according to a pseudo-random sequence. The maximum duration of each swim trial was 60 s. The location of the hidden platform remained constant from trial to trial. Thus, we were able to assess trial-independent spatial learning. A no-platform probe trial was also performed two hours after the completion of the single session hidden platform testing as a measure for reference memory. The platform in the probe trail was removed from the pool and the rats were allowed to swim freely for 30 seconds. The MWT environment was divided into four quadrants in which quadrants 1, 2, 3, and 4 were labelled for NE, SE (target quadrant in the current study), SW, and NW, respectively. The percentage of time that the animals spent in each quadrant of the task was recorded. Movements of the animals including latency to find the hidden platform, swim speed and error index (swim error or corridor percent time) were recorded and analyzed by an image-computerized tracking system (HVS Image 2020, UK). The error index in the present study refers to the accuracy of an animal’s swim trajectory within a 15-cm-wide corridor from the start point to the platform. Any deviation from this corridor during swimming was scored as an error^30^.

### Behavioural Procedures: Elevated Plus Maze (EPM)

Anxiety-related behaviour was assessed under dim illumination in the EPM three days before and 2 days after restraint stress (ref. 31 with modification) in the afternoon between 14:30 and 18:30 hours. The apparatus consisted of two open and two closed arms (all 85 × 17 cm) and was elevated 93 cm above the floor. The open arms had no side or end walls, and the closed arms had side and end walls (45 cm high). The rats (*N* = 10–12) were placed individually in the central square facing either left or right open arm, and were allowed to explore the maze for 8 min. The experimenter left the room immediately after placing the rat on the maze. Each animal was tested only once before and after stress, and the behaviour of the animals in the maze was recorded by a camera (CCTV Auto tracking PTZ; SONY, Tokyo, Japan) and analysed by a computer tracking system (SINA Motiongraph, V.II, 2011, Tabriz, Iran) through analysis of the standard measures (time spent in the arms, time spent in the end of the open arms, time spent in the central square) of the EPM. Path length (distance traveled), path speed, stop time (absence of forward movement for at least 1 s or greater) and dispersion (the distribution of stops on the table) were also analysed. In order to minimize olfactory cues, the apparatus was cleaned with 70% alcohol after testing each animal.

### Behavioural Procedures: Open-Field Table (OFT)

The OFT was used to assess depression-like behaviour^32^ in the rats 3 days after stress (morning between 8:00 and 11:30 hours; *N* = 7–10). The open-field table (a 200 cm diameter white circular table, elevated 80 cm above the floor^33^; with modifications) was used under dim illumination. A wooden cage (20 cm × 15 cm × 25 cm; transparent mesh ceiling) with a 9 cm × 9 cm entrance, facing the center of the table, was located at 12:00 o’clock on the table. Each rat was individually placed at 9:00 o’clock on the table and video recorded for 20 min with a camera mounted above the open field (CCTV Auto tracking PTZ; SONY, Tokyo, Japan). Video recordings were analysed for path length, path speed, stop time (bins of 1–3, 3–15, 15–35, 35 to < 60 and > 60 s indicated by bubble diameter) and dispersion (including in- and out-cage stops), average distance between stops, and time spent in the cage by the computer tracking system (SINA Motiongraph, V.II, 2011, Tabriz, Iran). Specifically, path length and path speed were measured as an indicator of motivation level^28^. After each animal, the apparatus was cleaned with 70% alcohol.

### Histological Analysis: Hippocampal Volumetry

Rats in all groups were randomly assigned to one of the histological and molecular assays due to the limited sample sizes. Two to four hours after behavioural testing, animals were euthanized as described previously^34^. To determine the hippocampal volume, a series of tissue sections (*N* = 5–6 per group) was stained with cresyl violet. The hippocampal volume in each rat was estimated according to the Cavalieri method^35, 36^ using a set of 10–11cross-sections of the hippocampal area, starting from −1.40 mm and terminating at −6.80 mm relative to bregma. In the case of missing or damaged sections (less than 10 sections per rat) data were calculated as the average area values from preceding and following sections.

### Molecular Analysis: *In Situ* Hybridization

Animals (*N* = 3–4 per group) were euthanized with an overdose of sodium pentobarbital and decapitated at the end of the experiment to rapidly remove the brains. All *in situ* hybridization experiments were carried out as previously described in detail (ref. 37 with modifications) and all sections were run under identical experimental conditions. Briefly, brains were sectioned with a cryostat (15 µm) approximately from −1.40 to −4.52 mm relative to bregma. The fixed, air-dried sections were incubated overnight with two 33P-labelled 48-mer oligonucleotide probes in hybridization buffer and the excess and unbound probe was washed off. Brain sections were exposed to BioMax MR-1 X-ray films for 4 weeks. Three to four comparable tissue sections per animal and region were considered for further analysis. Levels of brain-derived neurotrophic factor (BDNF) mRNA were analyzed and masked by optical densitometry of autoradiographic films using a computerized image analysis system (MCID, Canada) and ImageJ 1.49p (NIH, USA). BDNF mRNA data in this experiment are represented as percent of NMS-SH group.

### Molecular Analysis: BDNF Protein Analysis

The enzyme-linked immunosorbent assay (ELISA) was employed to quantify BDNF protein levels^37, 38^. Briefly, the tissues were homogenized in 100 × (w/v) ice-cold homogenization buffer containing a protease-inhibitor cocktail and further diluted 1:9 in this buffer to a total dilution of 1000×^37^. ELISA was performed on the homogenate using the BDNF Emax Immuno Assay Systems (Promega KK, Tokyo, Japan). Two brain areas of primary interest, the frontal cortex (~3.24 mm relative to bregma) and the hippocampus (HPC; ~−3.60 mm relative to bregma) were analysed for BDNF mRNA and protein (*N* = 3–4 per group).

### Adrenal Gland Weight

Adrenal glands were removed and weighed. The weights of the left and right adrenal glands were averaged and the absolute weight of adrenal glands was used for further analysis^39^.

### Statistical Analysis

The results were subject to analysis of variance (ANOVA; SPSS 16.0, SPSS Inc., USA). *Repeated measures* and *one-way* ANOVA, and dependent and independent sample *t*-tests were conducted when necessary. In the present study, *one-way* ANOVA was used because not all groups needed for a three-way ANOVA were available. Also, *post-hoc* test (Tukey) was used to adjust for multiple comparisons. In all cases, means of values were compared. Data for each behavioural test were analysed by separate analysis of ANOVA with a main between-subject factor of Group. Within-subject factors were designated for each test as appropriate, for example, Trial (1–12) and Quadrant (target/other quadrants) in Morris water task, Arm (open/closed arms, central square, end of open arms) for the elevated plus-maze, Stops (duration, in- or out-cage stops) for the open field table. Also, correlation coefficients were calculated to examine the correlation between plasma CORT levels, path speed in both dry- and wet-land tasks and HPC volume. Significance levels used for statistical tests were 0.05 and are denoted by one asterisk. Data are presented as means ± SEM.

## Results

### Social Isolation and Stress Synergistically Impact the Stress Response

#### Body Weight

A comparison between NMS and MS groups did not reveal a between-group effect (*p* = 0.063). When all groups were compared, however, repeated measures ANOVA indicated a significant main effect of Group (*F*_7,84_ = 8.71, *p* = 0.041) and a significant interaction between Group and Day (*p* = 0.044). Compared to other groups, only MS + S-SI and MS + S-SI + T groups failed to gain weight during stress sessions (all *p* ≤ 0.05, *post-hoc*; Fig. 1B). There were no differences in body weight before stress, however (*p* = 0.074). Post-stress changes in body weight, on the other hand, indicated a significant main effect of Group (*F*_7,83_ = 12.39, *p* = 0.044) showing all groups gained weight across 21 days except for MS + S-SI and MS + S-SI + T (all *p* ≤ 0.05, *post-hoc*). This indicates that the synergy of the adult stress and social isolation prevented body weight gain, and toy-playing experiences during stress did not alleviate the adverse outcomes of isolation housing.

#### Adrenal Gland Weight

In spite of an evident decrease in adrenal gland weight in MS compared to NMS animals (0.041 ± 0.011 g *vs*. 0.054 ± 0.01 g), no significant difference was observed between groups (*p* = 0.052). Two animals from NMS-SH, two animals from NMS + S-SI, one animal from MS-SI and two animals from MS + S-SI groups were excluded from this analysis due to damage to one or both adrenal glands.

#### Plasma CORT

**Figure 2:**
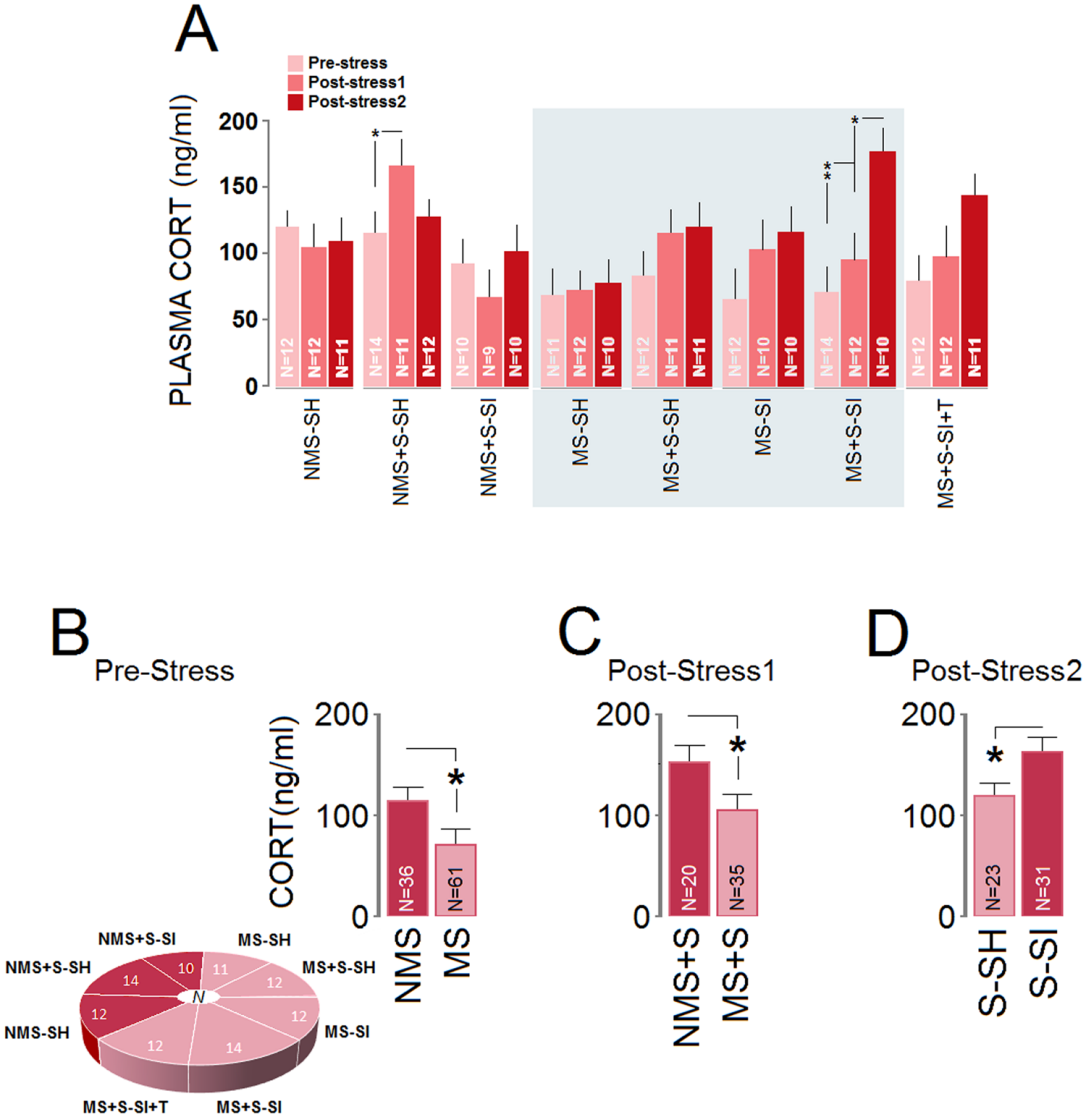
*Stress and social isolation exacerbate stress response in ancestrally stressed rats*. Shown are plasma corticosterone levels two days before stress (Pre-stress), the first day of stress (Post-stress1), and the last day of stress (Post-stress2). (**A**) Plasma CORT significantly rose in MS + S-SI rats in response to stress exposure only in the chronic phase. (**B**–**D**) Comparison of changes in CORT levels influenced by maternal multigenerational stress and the double-hit protocol at different time points. Asterisks indicate significant differences: *p ≤ 0.05, **p ≤ 0.01; ANOVA. Error bars show ± SEM.

Figure 2A–D illustrates circulating levels of CORT as assessed from blood samples. Seven animals in pre-stress assessment, 11 animals in post-stress 1 and 11 animals in post-stress 2 assessments in different groups were excluded from CORT analysis due to technical issues. (*i*) *Pre-stress*: Animals showed a significant overall effect of Group (*F*_1,94_ = 10.06, *p* = 0.048) with the NMS group displaying higher levels of CORT than the MS group (116.29 ± 13 ng/ml vs. 77.18 ± 15 ng/ml; panel **B**). (*ii*) *Post-stress1*: Increased plasma CORT levels were found in most adulthood stressed groups, especially NMS + S-SH animals on the first day (acute time point) of the adulthood stress. There was a significant effect of Group for CORT levels with the NMS + S rats demonstrating higher levels than the MS + S groups (163.82 ± 14 ng/ml vs. 104.82 ± 14 ng/ml*, F*_1,93_ = 9.33, *p* = 0.04; panel **C**). (*iii*) *Post-stress2*: A significant main effect of Group was observed in terms of CORT levels at the post-stress 2 time point (*F*_7,94_ = 11.68, *p* = 0.037) with the MS + S-SI and MS + S-SI + T groups showing higher CORT values than other groups (all *p* ≤ 0.05, *post-hoc*). Furthermore, S-SH and S-SI groups displayed a significant effect of Group (123.02 ± 11 ng/ml vs. 166.49 ± 12 ng/ml*, F*_1,52_ = 8.13, *p* = 0.041) with the S-SH rats showing lower levels of CORT than S-SI rats (panel **D**). Also, when compared with NMS + S-SI rats, the MS + S-SI group significantly displayed increased CORT levels at the post-stress 2 time point (*F*_1,18_ = 21.84, *p* = 0.03). In summary, changes in plasma CORT in the post-stress 2 phase indicated that social isolation during adulthood stress hampered the adaptive habituation response of the HPA system to chronic stress that was observed in social housing conditions.

### Histological Assessment

#### Hippocampal Volume

**Figure 3:**
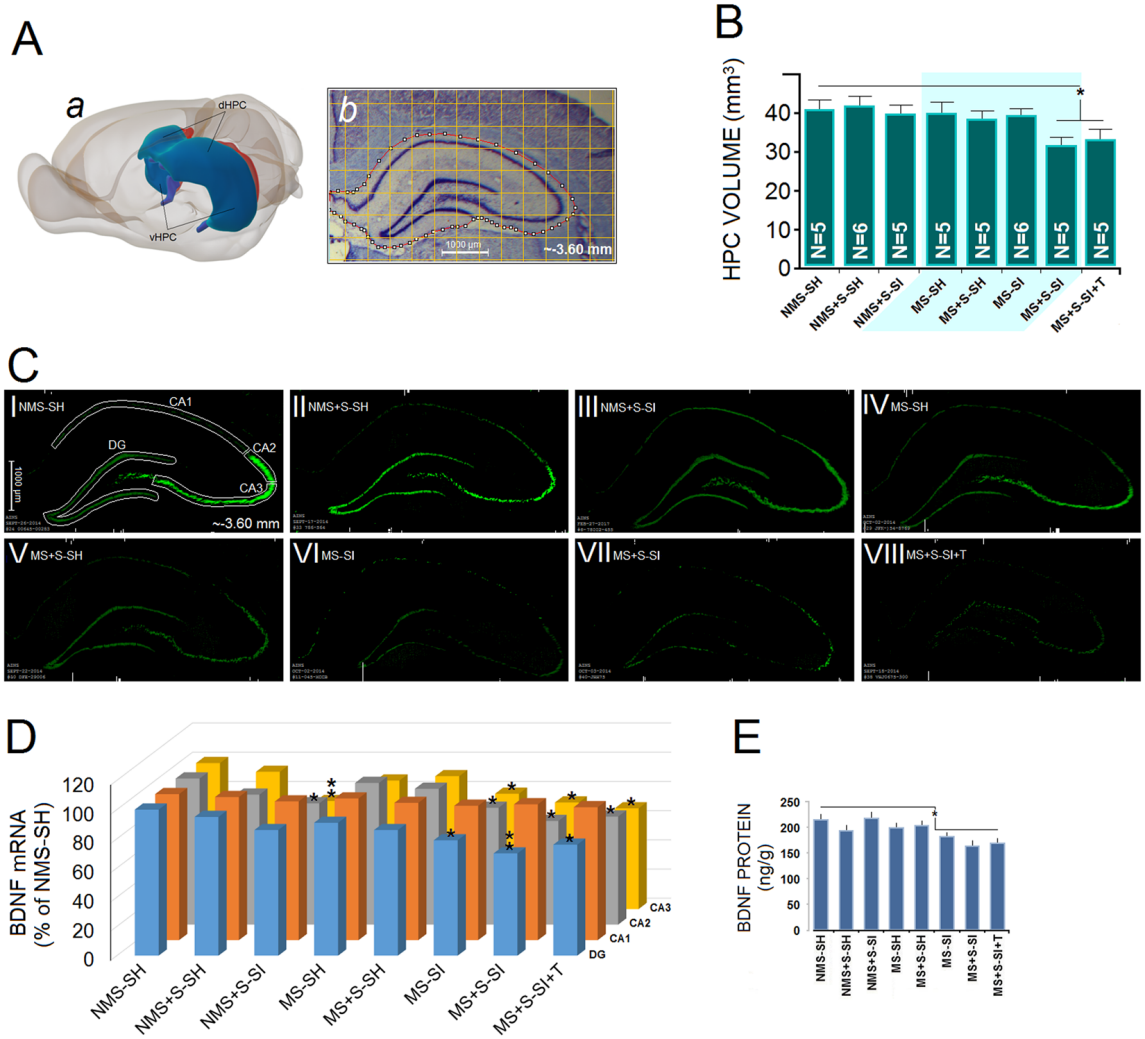
*Combined ancestral and adulthood stress reduce hippocampal volume and BDNF expression*. (**A**) A set of 10–11 cross sections of whole hippocampus (AP: −1.4 mm −6.80 mm) were considered for volumetric analysis. (*a*) 3D reconstruction of the dorsal and ventral hippocampus (blue) in the rat brain. (*b*) Coronal view of a left dorsal hippocampus illustrating the area that was considered for hippocampal volumetrics. (**B**) The MS + S-SI and MS + S-SI + T animals indicated decreased HPC volume when compared with other groups. (**C**) Subpanels I-VIII: The investigated anatomical subregions are delineated for NMS-SH animals. Autoradiographs (unsharp mask-HK1-RSZII2.60 filter-Green) of BDNF mRNA in the dHPC (CA1, CA2, CA3, and DG) indicated that only social isolation reduced BDNF mRNA expression in CA2, CA3 and DG in multigenerationally stressed animals. (**D**) Density of BDNF mRNA signal in CA1, CA2, CA3 and DG. BDNF mRNA data (*N* = 3–4 per group) in this experiment are represented as percent of the NMS-SH group; (**E**) BDNF protein levels in dHPC. No difference was observed between right and left dHPC in terms of BDNF mRNA expression and proteins. Asterisks indicate significant differences: *p ≤ 0.05, **p ≤ 0.01; ANOVA. Error bars show ± SEM.

Isolation during adulthood stress led to smaller HPC volume in both MS + S-SI and MS + S-SI + T groups compared to other MS and NMS groups (main effect of Group: *F*_7,34_ = 10.18, *p* = 0.041; Also, all *p* ≤ 0.05, *post-hoc*, Fig. 3A,B). A comparison between MS + S-SH and MS + S-SI groups indicated that socially secluded animals had lower HPC volume by 18%, and ~22% smaller HPC volume than the NMS + S-SH group. Furthermore, MS + S-SI and MS + S-SI + T rats showed approximately 20% smaller HPC volume when compared with NMS + S-SI rats. Thus, enrichment with toys during stress did not protect isolated animals against the aversive consequences of social isolation.

#### BDNF mRNA Expression

Although there were no changes in the frontal cortex (*p* = 0.34), *in situ* hybridization revealed that BDNF mRNA in the HPC was abundantly and differentially expressed in the DG, CA2 and CA3 but not in the CA1. A representative autoradiograph of the hippocampal BDNF mRNA expression from all experimental groups is shown in Fig. 3C). Most MS groups, especially animals subjected to adulthood stress and social isolation, showed a marked decrease in BDNF mRNA in the HPC sub-regions. A significant main effect of Group (*F*_7,18_ = 18.51, *p* = 0.042) and Region (*F*_3,18_ = 11.06, *p* = 0.04) but not Group by Region effect was observed in MS-SI, MS + S-SI and MS + S-SI + T animals based on prominent decrease in mRNA expression (Fig. 3D). When compared with NMS + S-SI, MS-SI and MS + S-SI + T groups, the MS + S-SI group indicated a significant decrease in mRNA expression in the DG and CA2 (main effect of region: *F*_3,9_ = 11.37, *p* = 0.036; all *p* ≤ 0.05, *post-hoc*) suggesting that social isolation during stress had a noticeable impact upon mRNA expression in the HPC sub-regions. Furthermore, MS + S-SI animals displayed less mRNA expression in the HPC sub-regions when compared to MS + S-SH (DG: ~16%; CA2: ~17%; CA3: ~12%) group.

#### BDNF Protein Expression

There was no significant between-group effect for BDNF protein levels in frontal cortex (*p* = 0.73). Also, in the HPC there was no difference between NMS (i.e. NMS-SH, NMS + S-SH and NMS + S-SI) groups despite a slightly increased level of HPC BDNF protein in NMS + S-SI (Fig. 3E). Therefore, an average of all NMS rats (205 ± 12 ng/g) was considered for baseline levels of the BDNF protein in the left and right HPC. ANOVA indicated no differences between right and left HPC in terms of BDNF proteins (*p* = 0.96). In all groups (except NMS + S-SI) subjected to social isolation, BDNF protein expression in the HPC was significantly decreased (168 ± 11.66 *vs*. 200.75 ± 11.75 ng/g; *F*_1,19_ = 11.18, *p* = 0.043) when compared with animals housed in pairs (standard housing). Although adulthood stress alone did not have a significant impact on hippocampal BDNF, adulthood stress and social isolation in multigenerationally stressed animals reduced BDNF proteins to about ~18% of basal levels.

### Social Isolation and Stress Impair Spatial Learning and Generate a Heightened Emotional State

#### Spatial Performance in MWT

**Figure 4:**
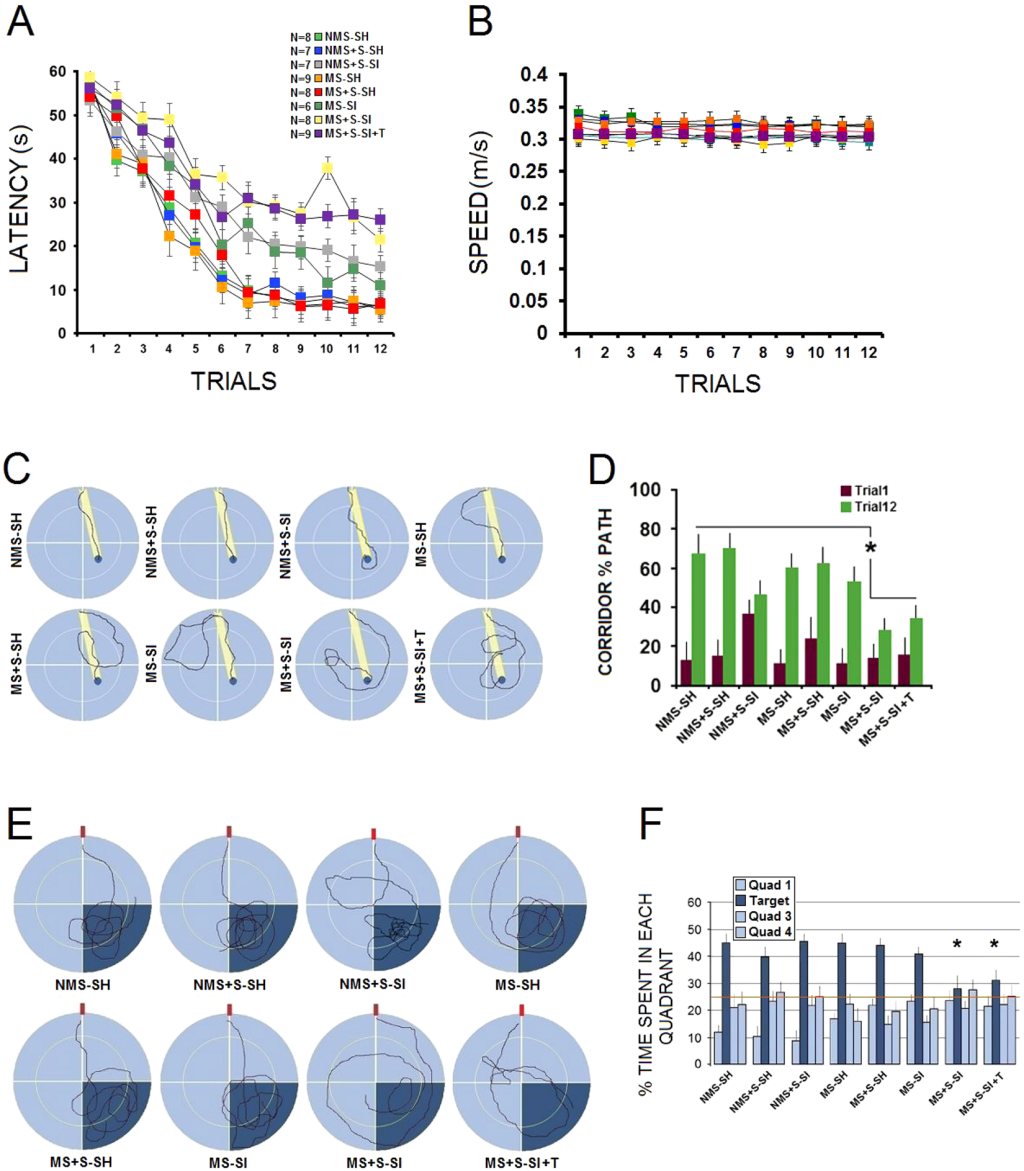
*Combined ancestral and adulthood stress impair spatial working and reference memory*. (**A**) Latency or time spent to find the hidden platform in the Morris water task using a one-day testing protocol and probe trial after stress. MS + S-SI and MS + S-SI + T rats displayed impaired spatial working memory when compared to controls. (**B**) Mean swim speed revealed no group differences. (**C**) Representative swim error in the last trial and (**D**) corridor percent time in the first and last trials indicating inaccurate swims relative to the platform location only in MS + S-SI and MS + S-SI + T rats. Yellow strips in plots indicate required swim corridors (Whishaw corridor) to the platform. (**E**) Representative probe trial trajectories illustrating focal search within the target quadrant in all groups except MS + S-SI and MS + S-SI + T rats. (**F**) The mean percentage of time spent in four quadrants of the task during the probe trial after completion of the single-session hidden platform paradigm. Rats in MS + S-SI and MS + S-SI + T groups showed no preference to spend time in the target quadrant (dark blue) indicating impaired reference memory. Asterisks indicate significant differences: *p ≤ 0.05; *Repeated-Measure* and *One-Way* ANOVA. Error bars show ± SEM.

Latency and swim length are standard indicators of successful spatial navigation in the MWT (Vorhees *et al*., 2004; Kapoor *et al*., 2009). Both, however, can be confounded by swimming speed. Here, only latency (time to find hidden platform) and speed are reported. A significant effect of Group (*N* = 6–9; *F*_7,54_ = 26.11, *p* = 0.042) and Trial (*F*_11,54_ = 17.91, *p* = 0.036) was found in terms of the latency in the MWT (Fig. 4A). Group × Trial effect was also significant (*p* = 0.033). *Post-hoc* comparison indicated that MS + S-SI and MS + S-SI + T rats spent more time (both *p* < 0.05) to find the hidden platform than any other group. Swim speed during the 12-trial-acquisition period showed a relatively flat speed line across 12 testing trials in all groups (Fig. 4B). The MS + S-SI rats navigated more slowly through the environment than other groups as testing proceeded. No significant difference, however, was observed between groups in terms of swim speed (*p* = 0.078).

Analysis of spatial error via corridor percent time in the MWT (Fig. 4C,D) indicated that MS + S-SI and MS + S-SI + T rats did not directly swim to the platform (*F*_7,54_ = 18.66; *post-hoc*: both *p* ≤ 0.05) when compared with other groups. This observation suggests inaccurate spatial navigation in multigenerationally stressed animals who experienced stress and social isolation during adulthood. A one-one way ANOVA also indicated that NMS + S-SI rats did not improve the percent time spent in the corridor when trial 1 and 12 were compared (*p* = 0.084). Moreover, probe trial (30-s duration, Fig. 4E,F) showed that rats in all groups displayed similar preference to spend time in the target quadrant (between 39.71–46.39%; quadrant two; SE) but MS + S-SI rats spent less time (27.98%). Thus, MS + S-SI rats arguably used a more diffuse pattern of searching, with much less spatial bias toward the former training quadrant when compared with other groups (all *p* ≤ 0.05, *post-hoc*). Examination of swim speed in the probe trial revealed no significant difference between groups.

Overall, results in the MWT indicate impaired working memory in the MS + S-SI and MS + S-SI + T rats. Dwell time in the quadrant that formerly contained the platform, also, was significantly lower for both groups relative to their controls (including NMS + S-SI) indicating an impaired reference memory. Hence, cognitive impairment in these groups became evident only after combined exposure to the two psychological adversities in adulthood.

#### Anxiety-related Behaviour in EPM

**Figure 5:**
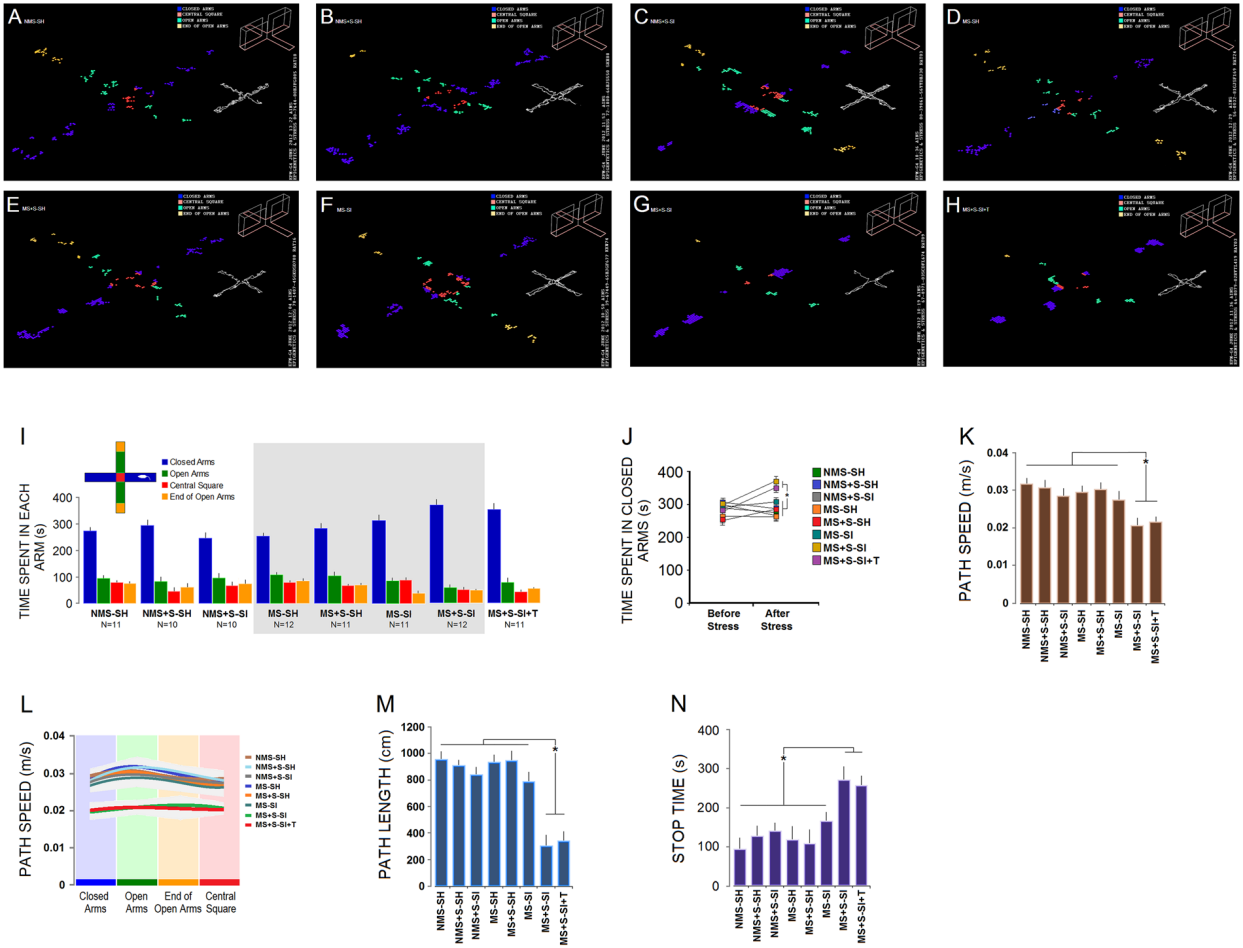
*Combined ancestral and adulthood stress induce an anxiety-like state*. (**A–H**) Dispersion of stops and paths made by rats in open and closed arms of the elevated plus maze shows different profiles of anxiety and motivation. (**G** & **H**) The MS + S-SI and MS + S-SI + T groups displayed higher anxiety and less motivation to explore the open arms than other groups. (**I** & **J**) MS + S-SI and MS + S-SI + T groups spent longer times in closed arms compared to other groups and compared to pre-stress session. They also explored the task with lower speed (**K** & **L**) associated with shorter path length (**M**) and more stop times (**N**) compared to other experimental groups. Asterisks indicate significant differences: *p ≤ 0.05; *One-Way* ANOVA. Error bars show ± SEM.

Emotionality and anxiety-related behaviours were examined in the EPM (Fig. 5A–N). In all measures, MS + S-SI and MS + S-SI + T spent more time in the closed arms (*F*_3,80_ = 23.59, *p = *0.036; *post-hoc*: both *p* ≤ 0.05; panels **I** & **J**), with lower speed (*F*_7,77_ = 15.37, *p* = 0.037; *post-hoc*: both *p* ≤ 0.05; panels **K** & **L**) and shorter path length (*F*_7,78_ = 23.07, *p* = 0.038; *post-hoc*: both *p* ≤ 0.05; panel **M**) suggesting that they displayed increased anxiety-related behaviours. Also, a significant effect of Arms (*F*_3,78_ = 26.93, *p* = 0.037) but no interaction between Group and Arms (*p* = 0.66) was found. MS + S-SI and MS + S-SI + T animals also spent less time in the open arms, an alternative index for anxiety in the EPM, and higher stop time (*F*_7,76_ = 11.39, *p* = 0.041; *post-hoc*: both *p* ≤ 0.05; panel **N**). These observations indicate a much greater anxiety-like response to the social isolation and adulthood stress in MS + S-SI and MS + S-SI + T animals than other experimental groups.

#### Depression-like Behaviour in OFT

**Figure 6:**
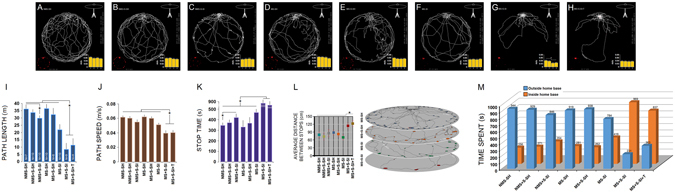
*Combined ancestral and adulthood stress alter the profile of exploratory behaviour*. (**A–H**) The paths taken by representative rats of different groups during a 20-min test session on the open field table. Note the length of path taken by MS + S-SI and MS + S-SI + T groups (**G** & **H**) that obviously are shorter than those by other groups. Home base was located at 12:00 O’clock on the table. (Bottom sub-panel-left; red dots) Representation of stops dispersion during exploration of the table top. (Bottom sub-panel-right) Rats’ speed during 20-min exploration assigned to four 5-minute intervals indicated that MS + S-SI and MS + S-SI + T groups were less motivated to explore the arena than other groups and made shorter path lengths (**I**) with lower speed (**J**) than other groups. Stop time (**K**) and average distance between stops (**L**) also indicated lower level of motor activity on the table in MS + S-SI rats compared to controls. Note the quadruple stack of plots that represent stops dispersion and distance between each stop during excursion. Stop duration is also represented by the bubble’s diameter. (**M**) Time spent inside or outside the home base by each group during a 20-minute session. MS + S-SI and MS + S-SI + T groups spent significantly more time inside the home base than outside, and more than any other groups.

Representative activity traces and parameters in the open-field are shown in Fig. 6A–M. ANOVA indicated that the distance traveled by the MS + S-SI and MS + S-SI + T rats over the 20-minute open-field session was significantly shorter than other groups (*F*_7,64_ = 18.39, *p* = 0.031; *post-hoc*: both *p* ≤ 0.05; panel **I**). As can be seen in panel I, the distance traveled by the MS + S-SI and MS + S-SI + T groups was equally short. No significant difference was found between the groups (*post-hoc*: *p* = 0.14). Furthermore, a separate one-way ANOVA conducted for path length in NMS groups (NMS-SH, NMS + S-SH and NMS + S-SI) showed a significant effect of Group (*F*_2,24_ = 10.49, *p* = 0.04) where only NMS + S-SI rats displayed shorter path length in the field when compared to NMS-SH animals (*post-hoc*: *p* ≤ 0.05; panel **I**). Because path length can be potentially affected by differences in path speed, both indices were considered complementary indicators of motivation in the OFT.

All groups displayed relatively constant speed (panel **J**) during free exploration on the table but the MS + S-SI and MS + S-SI + T groups (*F*_7,64_ = 27.49, *p* = 0.041; *post-hoc*: both *p* ≤ 0.05) exhibited significantly lower speed than other groups. Also, the increased duration of stops (stop time) in MS + S-SI and MS + S-SI + T rats (*F*_7,64_ = 18.12, *p* = 0.037; *post-hoc*: both *p* ≤ 0.05; panel **K**) indicated that adulthood stress along with social isoaltion reduced locomotor activity and exploration in the OFT, and that toy-playing experiences could not reduce the adverse motivational consequences of the loneliness during stress in multigenerationally stressed animals. Further analysis conducted with one-way ANOVA indicated that NMS + S-SI rats spent more times in stop during exploration compared to NMS-SH group (effect of Group:*F*_2,24_ = 8.52, *p* = 0.043; *post-hoc*: *p* ≤ 0.05; panel **K**). A significant main effect of Group was found in terms of the average distance between stops (*F*_7,63_ = 19.27, *p* = 0.038; *post-hoc*: both *p* ≤ 0.05; panel **L**) suggesting that lower speed in the MS + S-SI and MS + S-SI + T groups was also associated with fewer stops during excursion than other groups.

Stop duration measured for inside or outside the home cage on the OFT served as an additional indicator of free exploration. Again, a main effect of Group (*F*_7,65_ = 22.91, *p* = 0.03; panel **M**) associated with significant effect of In-Out (*F*_1,65_ = 37.16, *p* = 0.01) was observed. Thus, MS + S-SI and MS + S-SI + T groups spent more time inside the home cage when compared with other groups (*post-hoc*: both *p* ≤ 0.05). These data suggest that adulthood stress associated with loneliness in multigenerationally stressed rats promotes behaviours that may be implicated in the development of depression-like behaviours.

### Correlation Assessment

Correlations between neurohormonal and motivational responses revealed a significant negative correlation between CORT levels in post-stress 2 and path speed in EPM (*r* = −0.851, *p* = 0.002) and OFT (*r* = −0.797, *p* = 0.010) but not MWT (*r* = 0.578, *p* = .133) only in MS + S-SI rats (*N* = 8–10). Thus, HPA axis hyperactivity was associated with a motivational deficit only within dry-land tasks. Furthermore, CORT levels of post-stress 2 correlated negatively with HPC volume (*r* = −0.721, *p* = 0.019) in the MS + S-SI group which suggests that high CORT values were associated with smaller hippocampal volume. No other significant correlations were found (data not shown).

## Discussion

The present study provides the first account of synergistic interactions between two adverse events in female rats with a history of four generations of multigenerational stress. The major findings of this study include the following: (1) gestational prenatal stress can induce cumulative effects that remain silent across four generations and (2) generates a phenotype that is vulnerable to a depression-like state in adulthood when challenged with stress; (3) neither social isolation nor adulthood stress individually unmask the latent neuro-behavioural consequences of ancestral gestational stress in F4 rats; however (4) F4 stressed rats that were socially isolated during adulthood stress displayed dysregulation of HPA-axis activity, hippocampal atrophy and psychomotor retardation, a delayed onset of depression-relevant changes induced by synergy between two hits of stress.

The present study addressed the effects of ancestral stress in interaction with stress in later life by using the physiological measures, such as body and adrenal gland weight as well as plasma CORT level. Stress exposure across four generations did not affect body and adrenal gland weight but the CORT level: the basal plasma value in MS rats was significantly lower than in NMS rats. The blunted basal CORT along with unaffected emotionality after prenatal stress was previously reported in female rats and linked to sex-specific genetic and epigenetic regulation^40^. From a psychopathological view, multigenerational stress may lead to blunted responsiveness of the HPA axis to recurrent stress and sensitize individuals to psychiatric disturbances, thus mimicking hypocortisolism, a characteristic maladaptive neurohormonal stress response that predicts risk of post-traumatic stress disorder (PTSD)^41^.

Also, multigenerational stress may down-regulate the HPA axis activity to the level of what is commonly referred to as stress resilience when challenged with recurrent stress^42, 43^. This response represents a dynamic process activated by a potentially traumatizing prolonged event^44, 45^ or even an active coping response^46^ to ancestral recurrent adversities. Hence, our data indicate that ancestral adverse experiences enhance stress tolerance in the subsequent offspring and promote resilience across four successive generations, although HPA-axis dysregulation and/or adaptive mechanisms that might contribute to resilience are still not completely understood^47^.

When exposed to the second hit by stress in adulthood, ancestrally stressed animals showed an exacerbated response of the HPA axis that abolished resilience. Consequently, CORT levels in the MS + S-SI rats remained significantly elevated at the chronic time point relative to its values at the pre-stress level. Similar chronic glucocorticoid elevations caused by impaired HPA negative feedback have previously been linked to depression in humans^48^ and depressive-like behaviours in animals^49^. By contrast, NMS or even MS rats challenged with adulthood stress but housed in pairs during stress were able to return to their pre-stress CORT values and habituate in terms of HPA axis activity^50^.

The present observations support the concept of a two-hit hypothesis^25, 26^ proposing that females become vulnerable to altered affective states, such as anxiety, after exposure to recurrent stress^51^. Heightened vulnerability to disease may occur through cumulative wear and tear by stress, according to the concept of allostatic load^52, 53^. Body weight, on the other hand, displayed an alternative profile of adaptation; only MS + S-SI rats failed to gain weight influenced by the cumulative effects of stress and social isolation. Here, stress-induced inhibition of food intake resembles those described earlier^54, 55^, and corticotropin-releasing hormone (CRH) released and neuropeptide Y (NPY) secreted by the hypothalamus during stress have been shown to impede food intake and inhibit body weight gain^56, 57^.

In addition, combined exposure to stress and isolation challenges in F4 stressed rats also induced HPC atrophy and dysfunction in the absence of detectable adverse effects on the frontal cortex, although both structures are sensitive to chronic stress exposure^58–60^. More importantly, dysfunction of these brain regions has been reported in depression^61^. The HPC, specifically, seems the most widely investigated structure in relation to depression because it shows the highest density of corticosteroid receptors in the brain^62^ and physiologically is closely connected to the inhibitory feedback in the HPA system^63, 64^. Here, HPC atrophy and decreased BDNF mRNA and protein expression in ancestrally stressed rats supports the double-hit hypothesis and provides further evidence that reduced HPC volume may be a trait characteristic for depression^65^. Further quantitative BDNF analyses and evaluations of HPC neuroanatomy would be required to provide additional mechanistic links. It should be noted, however, that changes in hippocampal spatial function must not be defined merely based upon HPA-axis dysregulation or elevated plasma CORT^66, 67^ even though CORT might still be causally involved in such alterations.

In addition to cognitive impairments, social isolation during adulthood stress in F4 ancestrally stressed rats triggered vulnerability to depressive-like behaviours. F4 stressed rats who were socially isolated during stress displayed motivational deficits (e.g. decreased path length and speed, and enhanced stop time^68, 69^ and more avoidance behaviours^33^ reflected by increased time spent in the closed arms in the EPM and inside the home cage within the OFT). These inhibitory effects on exploratory behaviours may be linked to high emotionality (anxiety and fear), fatigue and behavioural despair^70^.

The eight-group design of the present study allows dissociation of motivational and emotional changes in multigenerationally stressed animals based on single- vs. double-hit paradigms. The design confirms that only a synergy between adulthood stress and social isolation in F4 stressed rats can articulate the latent vulnerability to behavioural deficits and alter the normal profile of motivation related to depression with a sustained effect on body weight in adulthood. Maladaptive neurohormonal responses may also indicate that ancestrally stressed rats experienced high emotionality associated with the lack of social support during isolation. Therefore, the present double-hit protocol not only confirms an impaired adaptive habituation indicated by the lasting CORT elevation but also greater psychomotor inhibition shown by the failure to gain weight in ancestrally stressed rats.

The observation that social support mitigates the adverse consequences of stress is in agreement with earlier findings. A recent study by Seetharaman and others^71^ indicated that daily exposure to a social environment reduces the risk of PTSD-like effects in rats. The authors suggested that social stimulation may reduce traumatic memories and therefore lead to reduced fear-related behaviour^71^. In addition, social support is particularly effective in reducing PTSD risk, chronic anxiety and stress^72^ while the lack thereof is associated with greater stress response and PTSD risk^73, 74^. In addition, interventions that provide social support can reduce perceived stress during pregnancy^75^, improve early brain development^76^ and mental and cognitive health^77^. The present experimental data not only support these findings, but suggest that social stability and support may alleviate the transgenerational transmission of stress and thus potentially improve mental health outcomes.

The present results also linked the effect of stress to structural changes and synaptic connectivity in association regions of the brain (e.g. HPC) and animal behaviour, although the effects of multigenerational stress in brain circuits are not well understood^78^. We believe repeated functional assessment using optogenetic^79^ and imaging tools^80–82^ combined with animal behaviour is ideally suited to monitor in real time the process by which multigenerational stress changes the structure and function of different brain circuits.
